# A new species of Numbakullidae Guţu & Heard, 2002 (Tanaidacea, Peracarida, Crustacea) from the Great Barrier Reef, Australia

**DOI:** 10.3897/zookeys.337.5903

**Published:** 2013-10-01

**Authors:** Anna Stępień

**Affiliations:** 1 Department of Invertebrate Zoology and Hydrobiology, University of Łódź, Banacha 12/16, Łódź 90-237, Poland

**Keywords:** Tanaidacea, Numbakullidae, *Numbakulla*, Australia, CReefs, coral reefs

## Abstract

A new species of *Numbakulla* Guţu & Heard, 2002 (Tanaidacea) is described from Heron Island (southern Great Barrier Reef, Queensland) collected during the Census of Coral Reefs Ecosystem (CReefs) program. The new species is the third member of the family and can be recognized by the combination of characters as: length/width ratio of the body, which is 6:7, pereonite 4 longer than the rest, the presence of eyes, a blunt rostrum, antenna article 2 elongated, cheliped carpus with row of inner setae, pereopod 6 carpus with spines, pleopod endopod with denticles.

## Introduction

To date 176 species of Tanaidacea have been described from Australia. The best studied parts of the continent are the Bass Strait (Victoria) with 66 species (Błażewicz-Paszkowycz and Bamber 2007a, b, 2012; Bamber and Błażewicz-Paszkowycz 2013), Moreton Bay (Queensland) with 35 species (Boesch 1973; Guţu 2006a; Błażewicz-Paszkowycz and Bamber 2007a, b; Bamber 2008, 2013), and Esperance Bay (Western Australia) with 24 species (Bamber 2005). In contrast, the tropical coast of Australia with its coral reefs is comparatively poorly studied, with only 14 species of Tanaidacea described.

This paper describes a new species of *Numbakulla* Guţu & Heard, 2002 from the Great Barrier Reef (eastern Australia). The material was collected in the vicinity of Heron Island during the Census of Coral Reefs Ecosystem (CReefs) program in 2010.

*Numbakulla* Guţu & Heard, 2002 was erected for two species *Numbakulla pygmaeus* Guţu & Heard, 2002 (type species, continental shelf of north-western Australia) and *Numbakulla srilankensis* Guţu, 2006 (coral reefs of Sri Lanka), and was defined principally by the presence of filtering setae on the merus and carpus of pereopod 1 and the longer third article of the maxilliped. These characters, together with the large carapace and pleotelson, pereopod 6 differing from pereopods 4–5, and the presence of only four pairs of pleopods were considered of sufficient significance to create the new family, the Numbakullidae.

## Material and methods

Material was collected during the CReefs expeditions organized by AIMS (Australian Institute of Marine Science) to Heron Island (Great Barrier Reef) in 2010. Pieces of coral rubble were collected by hand while SCUBA diving and placed into buckets (20 l) with a few drops of formaldehyde to encourage animals to leave their microhabitats (tubes and crevices). The samples, with the animals still alive, were then washed over a fine mesh (0.3 mm), the residue sorted under the microscope, and tanaidacean specimens were collected and preserved in 80% ethanol (see Stępień and Błażewicz-Paszkowycz 2009). Material is held at the Museum of Tropical Queensland, Australia (MTQ).

Morphological terminology follows Błażewicz-Paszkowycz and Bamber (2012).

## Results

### Systematics
Suborder Apseudomorpha Sieg, 1980

#### Family Numbakullidae

Guţu & Heard, 2002

http://species-id.net/wiki/Numbakullidae

##### Remarks.

Guţu and Heard (2002) mentioned some morphological similarities between Numbakullidae and two other families: Kalliapseudidae Lang, 1956 and Metapseudidae Lang, 1970. The presence of filtering setae suggests a relationship to Kalliapseudidae. Guţu and Heard (2002) also listed number of characters between the two families, which imply that present of setae is a parallelism.

Numbakullidae resembles the Metapseudidae in the appearance of pereopods 2–5 (elongated propodus and well-developed dactylus). Guţu (2006) later suggested that *Numbakulla* is most closely similar to three genera from subfamily Chondropodinae Guţu, 2008 (Metapseudidae): *Calozodion* Gardiner, 1973, *Zaraza* Guţu, 2006c and *Chondropodus* Guţu, 2006a based on configuration of terminal lobe of labium.

Despite this similarities there are many characters which distinguish the Chondropodinae from the Numbakullidae: short pleotelson, usually with lateral incision, spines on antennule, labium with lateral spines, pereopod 1 digging type, with row of spines and plumose setae on basis, and propodus longer than carpus. At this point, therefore the relationships of the Numbakullidae remained unclear.

Undoubtedly *Numbakulla* represents a separate family. According to a phylogenetic analysis based on morphological characters, the Numbakullidae is not closely related to either the Kalliapseudidae nor the Metapseudidae (personal observations, study in progress).

#### Genus Numbakulla

Guţu & Heard, 2002

http://species-id.net/wiki/Numbakulla

##### Diagnosis.

(amended after Guţu and Heard 2002): Body dorso-ventrally flattened, elongated, more than four times as long as wide. Cephalothorax and pleotelson very large in relation to pereon and pleon (cephalothorax at least as long as first three pereonites combined, pleotelson as long as all pleonites combined). Pereonites and pleonites wide and short, all similar in length or fourth pereonite longer than the rest, pleonites all similar in length. Eyes present or absent. Maxilliped palp with numerous inner plumose setae. Epignath elongate, with long terminal spine. Cheliped with exopodite, carpus and propodus very large. Pereopod 1 with exopodite bearing distally 4 to 5 plumose setae, propodus very short, at least two times shorter than carpus. Pereopod 6 with long carpus and propodus and with long plumose setae on basis, merus and carpus. Four pairs of pleopods. Uropod with elongated basis and multisegmented endopod, inserted medially on lateral margin of pleotelson. Sexual dimorphism very pronounced in large size of male cheliped.

##### Type species.

*Numbakulla pygameus* Guţu & Heard, 2002, by monotypy.

##### Species included.

*Numbakulla pygameus* Guţu & Heard, 2002, *Numbakulla srilankensis* Guţu, 2006, *Numbakulla pii* sp. n.

#### Numbakulla pii sp. n.

http://zoobank.org/34385B01-7446-45DA-863D-A22C97B1B808

http://species-id.net/wiki/Numbakulla_pii

Figs 1 –4 , Photo 1

##### Material examined.

*Holotype*: female (MTQ W34252), Stn HI 10-009E, 23°25'53"S, 152°2'57"E, Sykes Reef, reef slope, coral rubble on sand, 12m depth, 14 November 2010, coll. C. Buxton.

*Paratypes*: 14 females (MTQ W34253), Stn HI 10-009E, same locality as holotype; eleven females (MTQ W34254), Stn HI 10-009D, 23°25'53"S, 152°2'57"E, Sykes Reef, reef flat, small coral rubble on sand, 12 m depth, 14 November 2010, coll. M. Błażewicz-Paszkowycz, C. Buxton; one female (MTQ W34256) Stn HI 10-009B, 23°25'53"S, 152°2'57"E, Sykes Reef, reef slope, coral rubble at base of the wall, 27 m depth, 14 November 2010, coll. S. Smith, C. Buxton; four females (MTQ W34255) Stn HI 10-013A, 23°35'12"S, 152°3'44"E, Lamont Reef, reef slope, coral rubble on sand, 21 m depth, 15 November 2010, coll. C. Buxton.

##### Etymology.

Named after Pi Patel, the central character from the novel Life of Pi, written by Yann Martel, one of favourite authors novel.

##### Diagnosis.

Body dorsoventrally flattened, 6.7 times longer than wide, blunt rostrum, eyes present, pereonite 4 clearly longer than the rest, mandible palp articles 2 with spines on outer margin, lacina mobilis narrow, one-denticled, antennule outer flagellum with six segments, antenna peduncle article 2 elongated, cheliped carpus with inner row of seven setae, pereopod 1 basis with row of setae, pereopod 6 carpus with row of ventral spines, pleopod endopod with proximal acute denticles, uropod endopod with 13 segments.

##### Description of female.

**Photo 1.**
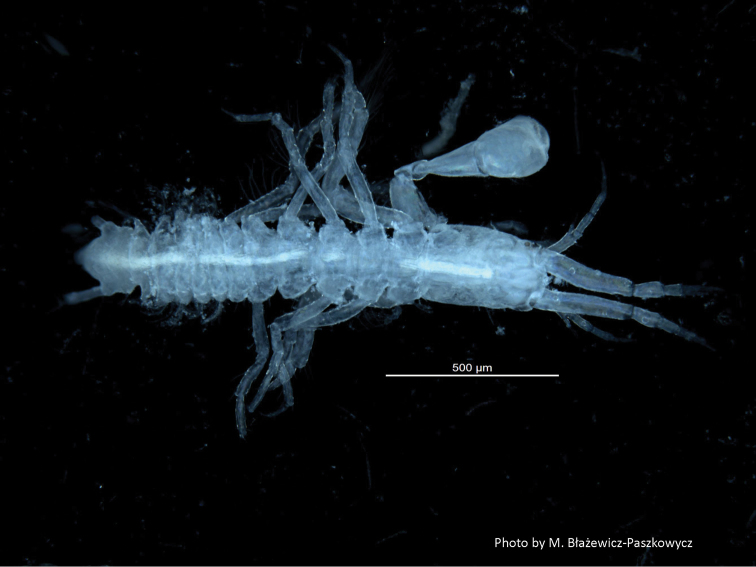
*Numbakulla pii* sp. n., paratype female. Body dorsal view.

**Figure 1.**
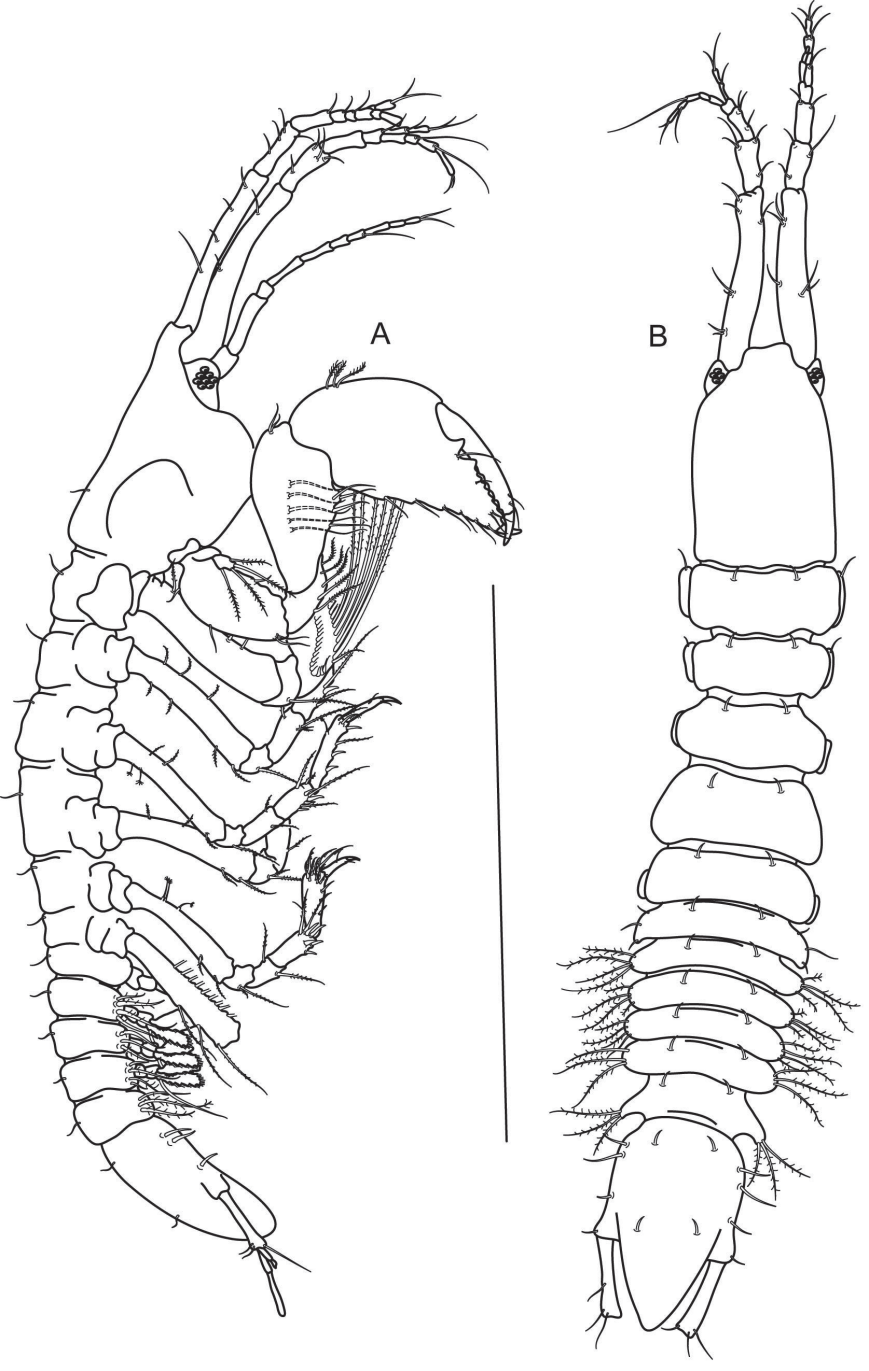
*Numbakulla pii* sp. n., holotype female. **A** body dorsal view **B** body lateral view. Scale line = 1 mm.

Body (Figs 1A, B, Photo 1) 1.7 mm long, 6.7 times as long as wide. Cephalothorax 22% of total body length, with blunt rostrum; ocular lobes present, with visual elements. Pereon: pereonites 1, 2 and 3 similar in length, 0.3 times as long as wide, pereonite 4 longest, 0.4 times as long as wide; pereonites 5 and 6 shorter, about 0.2 times as long as wide; all pereonites with pair of setae dorsally; last pereonite with three setae laterally. Pleon 19% of total body length, first four pleonites similar in length, 0.1 times as long as wide, with three or four long plumose setae laterally and pair of setae dorsally, and with rounded lateral margin; last pleonite 0.2 times as long as wide, with three plumose setae laterally and acute lateral margin. Pleotelson acute posteriorly, 1.6 times as long, with four setae laterally and two pairs of setae dorsally.

**Figure 2.**
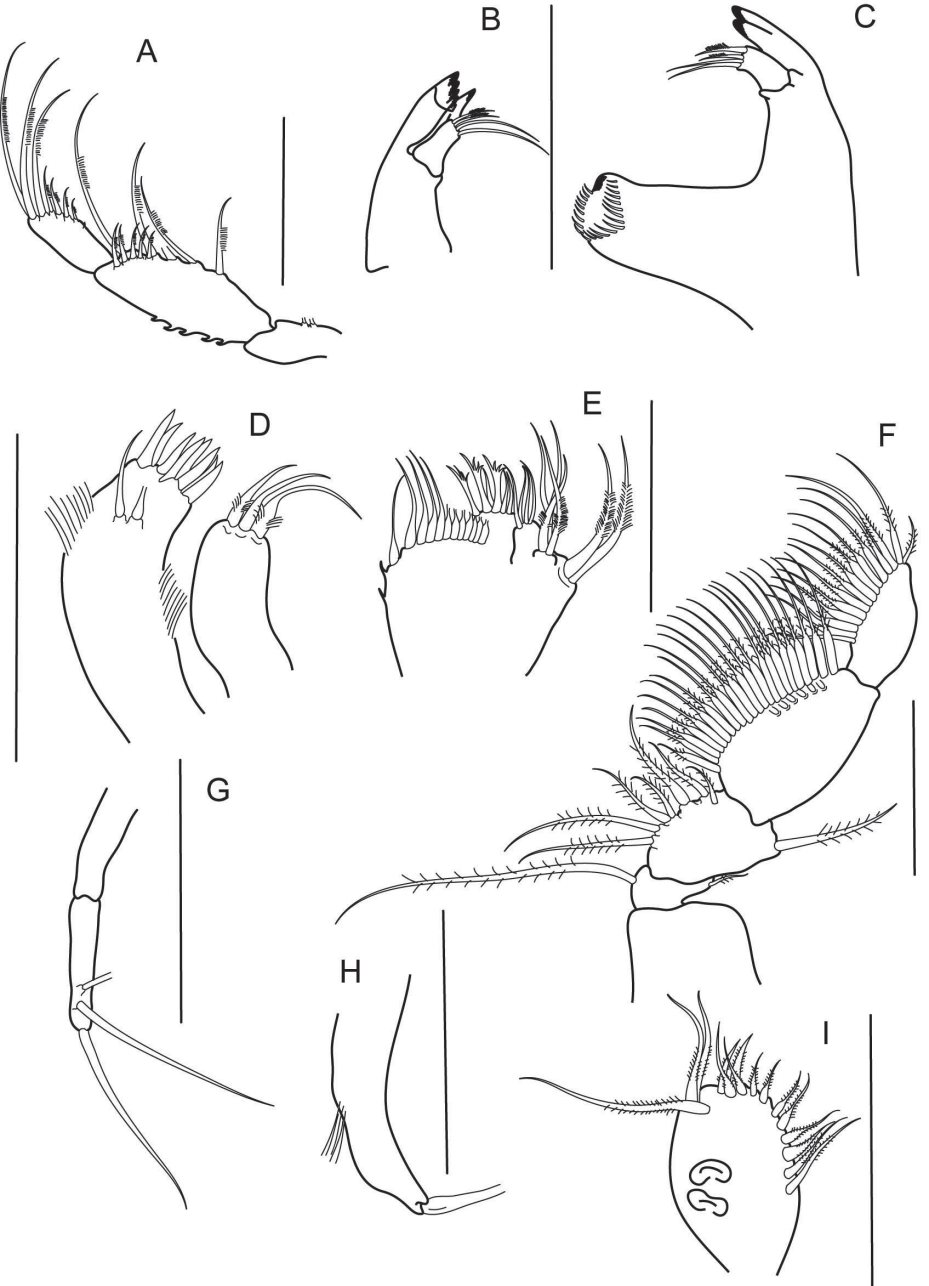
*Numbakulla pii* sp. n., **A** palp of left mandible **B** left mandible **C** right mandible **D** maxillule **E** maxilla **F** maxilliped **G** palp of maxillule **H** epignath **I** maxillipedal endite. Scale line = 0.1mm.

**Figure 3.**
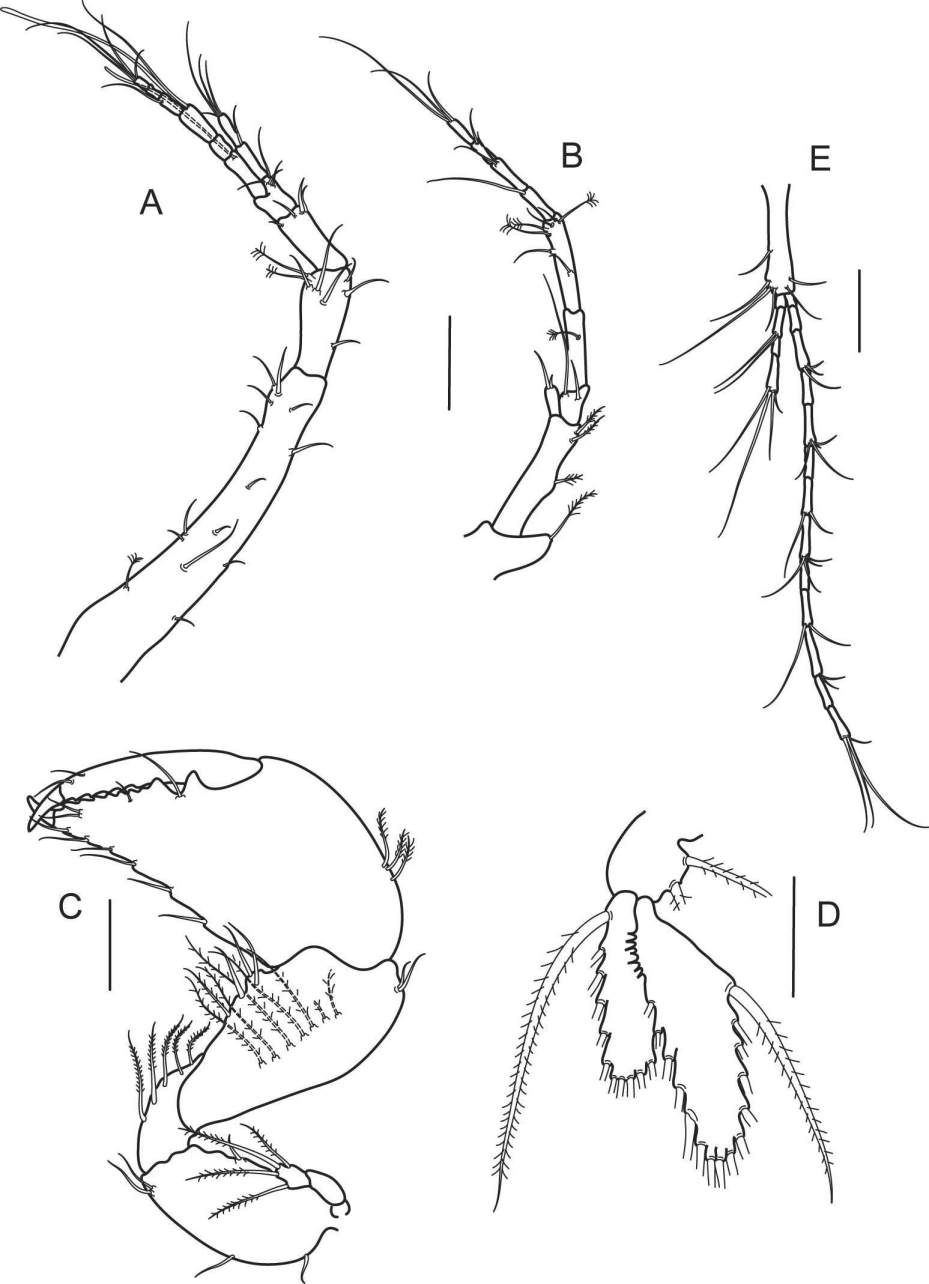
*Numbakulla pii* sp. n., **A** antennule **B** antenna **C** left cheliped **D** pleopod **E** uropod. Scale line = 0.1 mm.

Antennule (Fig. 3A): peduncle article 1 7.5 times as long as wide, with one penicillate seta on outer margin, and with 13 simple setae on whole surface; article 2 0.2 times as long as article 1, with one simple seta on inner margin and with five simple setae and two penicillate setae on distal margin; article 3 2.4 times as long as wide, with four distal simple setae; article 4 with four distal simple setae; outer flagellum with six segments, segments 1 and 3 with aesthetasc, last segment with four simple setae; inner flagellum with two segments, last segment with four simple setae.

Antenna (Fig. 3B): peduncle article 1 with rounded apophysis bearing distal plumose seta; article 2 elongate, five times as long as wide, with three inner plumose setae; squama narrow, bearing two simple setae; article 3 0.2 times as long as article 2, with two distal simple setae; article 4 0.6 times as long as article 2, with one penicillate seta; article 5 similar in length to article 4, with two mid-length simple setae, and distally with two simple, and three penicillate setae; flagellum with four segments, distal segment with three simple setae.

Mouthparts: Left mandible (Fig. 2B) bearing denticulate pars incisiva; lacinia mobilis with one blunt tooth; setiferous lobe with two simple and two plumose setae; palp (Fig. 2A) article 1 1.7 times as long as wide, with two inner setae; article 2 2.5 times as long as wide, with five long and five short plumose setae on inner margin, and with denticulate outer margin; article 3 2.2 times as long as wide, with seven inner plumose setae. Right mandible (Fig. 2C) with two teeth on pars incisiva; pars molaris robust, margin with row of simple setae. Maxillule (Fig. 2D) outer endite with eight distal spines and two subdistal setae; inner and outer margin finely setose; inner endite with three distal plumose setae; palp (Fig. 2G) two-articled, with two subdistal and one distal setae. Maxilla (Fig. 2E) outer lobe of moveable endite with plumose setae, two subdistally and four on distal margin; inner lobe with four distal simple setae; outer lobe of fixed endite with some simple setae and three trifurcated setae; inner endite with rostral row of twelve setae, inner margin with two denticles. Maxilliped (Fig. 2F) basis 0.8 times as long as wide, naked; palp article 1 0.4 times as long as wide, with one outer plumose seta and with one long inner plumose setae; palp article 2 1.3 times as long as wide, with one outer plumose seta, and with inner row of seven plumose setae; palp article 3 1.7 times as long as wide, with two inner rows of plumose setae (marginal row of 23 setae, submarginal row of six setae); palp article 4 2.7 times as long as wide, with inner row of 15 plumose setae. Endite (Fig. 2I) with 15 plumose setae along outer and distal margin, and with two coupling hooks. Epignath (Fig. 2H) narrow, with strong distal seta.

Cheliped (Fig. 3C) basis 1.8 times as long as wide, ventrally with two proximal setae, and two distal setae, and dorsally with one seta; exopodite three-articled, last article with four plumose setae; merus 1.5 times as long as wide, with five ventral plumose setae; carpus 1.7 times as long as wide, with four ventrodistal setae, two dorsodistal setae, and with row of seven inner plumose setae; palm of propodus as long as wide, with three dorsal plumose setae and with six simple setae along ventral margin of palm and fixed finger; fixed finger as long as palm, cutting edge crenulated, with four simple setae, and two proximal tooth-like apophysis; dactylus with unguis 1.2 times as long as propodus, with two dorsal setae, cutting edge crenulated.

**Figure 4.**
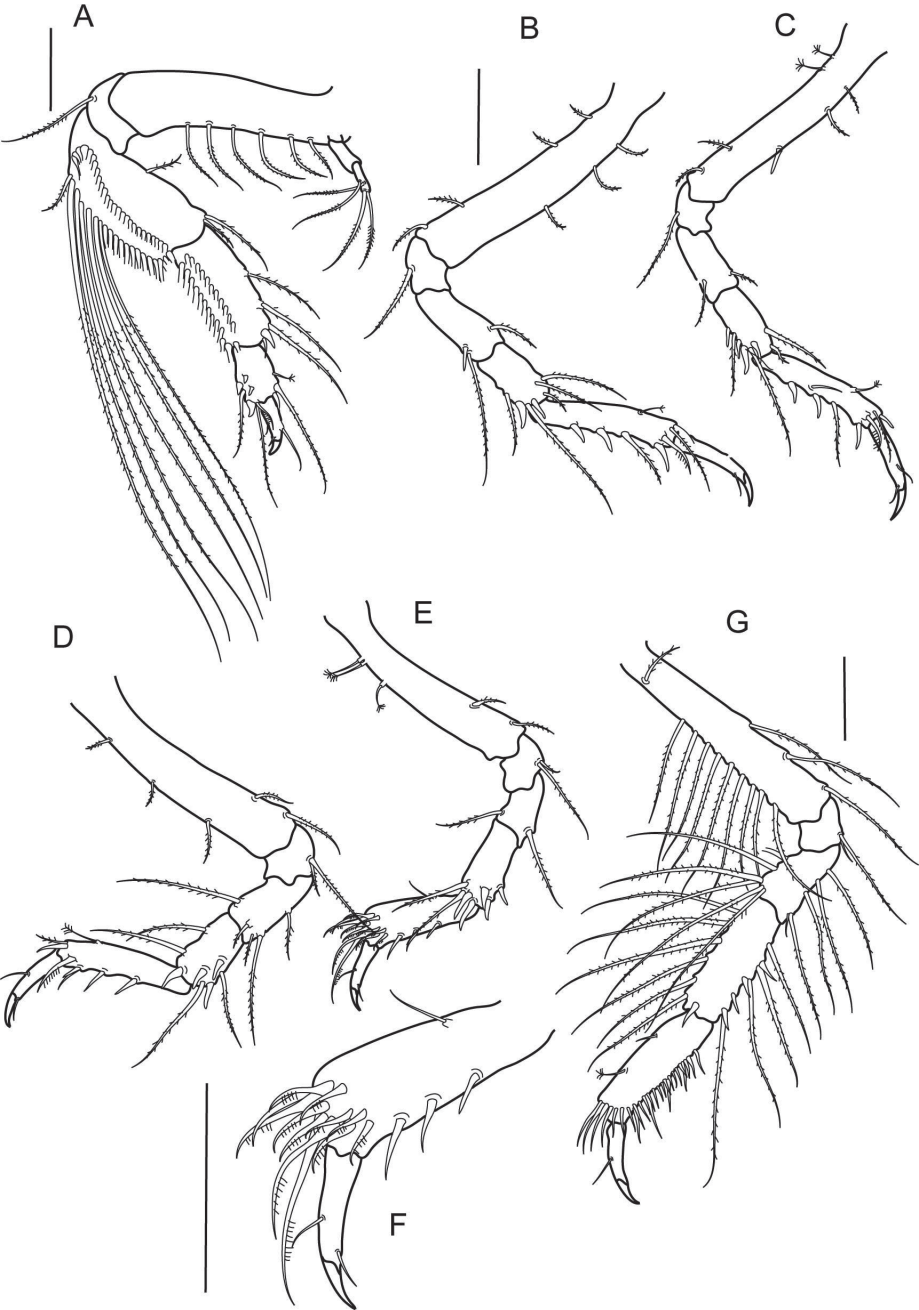
*Numbakulla pii* sp. n., **A** pereopod 1 **B** pereopod 2 **C** pereopod 3 **D** pereopod 4 **E** pereopod 5 **F** propodus of pereopod 5 **G** pereopod 6. Scale line = 0.1 mm.

Pereopod 1 (Fig. 4A) basis four times as long as wide, dorsally with six plumose setae; exopodite three-articled, distal article with four plumose setae; ischium 0.3 times as long as wide, with one ventrodistal seta; merus 1.8 times as long as wide, with mid-dorsal plumose seta and two dorsodistal plumose setae, ventral margin with two rows of numerous, long plumose setae; carpus 0.7 times as long as merus, dorsally with three plumose setae, one simple setae and one spine, ventral margin with two rows of numerous plumose setae; propodus 1.3 times as long as wide, half as long as carpus, with one penicillate and one plumose setae, and one spine dorsally, ventral margin with three plumose setae and four spines; dactylus together with unguis as long as propodus, with one dorsal, and two ventral setae.

Pereopod 2 (Fig. 4B) basis six times as long as wide, with four ventral plumose setae and three dorsal setae, ischium as long as wide, with plumose ventral seta; merus three times as long as wide, ventral margin with one plumose seta and thin spine, dorsal margin with one plumose seta; carpus 0.7 times as long as merus, with two plumose setae and two spines ventrally, and with three dorsodistal plumose setae; propodus 5.3 times as long as wide, 2.1 times as long as carpus, with one dorsal penicillate setae, ventral margin with one plumose seta and three spines, three finely denticulate setae near dactylus insertion; dactylus together with unguis 0.7 times as long as propodus, with seta on dorsal and ventral margin.

Pereopod 3 (Fig. 4C) similar to pereopod 2, but basis with two ventral penicillate setae.

Pereopod 4 (Fig. 4D) basis 5.6 times as long as wide, with three dorsal plumose setae and with two ventral plumose setae; ischium 0.6 times as long as wide, with two ventrodistal plumose setae; merus 1.6 times as long as wide, with dorsal plumose seta and with three dorsodistal and one mid-dorsal plumose setae; carpus 1.1 times as long as merus, with two dorsal plumose setae, and with four spines and two plumose setae on ventrodistal and distal margin; propodus 4.8 times as long as wide, 1.7 times as long as carpus, with dorsal penicillate seta, ventral margin with three thin spines and one simple seta, and three finely denticulated setae near dactylus insertion; dactylus together with unguis 0.8 times as long as propodus, with simple seta on ventral and dorsal margin.

Pereopod 5 (Fig. 4E) basis 5.5 times as long as wide, with three proximal penicillate setae on dorsal margin and with two ventral plumose setae; ischium 0.9 times as long as wide, with two plumose ventrodistal setae; merus 1.3 times as long as wide, with plumose seta dorso- and ventrodistally; carpus 1.6 times as long as merus, with five spines and two plumose setae on distal margin; propodus (Fig. 4F) three times as long as wide, 1.3 times as long as carpus, with three thin ventral spines, and with eleven finely denticulate plumose setae near dactylus insertion; dactylus together with unguis 0.7 times as long as propodus, with simple setae on dorsal and ventral margin.

Pereopod 6 (Fig. 4G) basis 6.8 times as long as wide, with ten dorsal plumose setae, and with three ventral plumose setae; ischium 0.6 times as long as wide, with two ventrodistal plumose setae; merus 1.7 times as long as wide, with six dorsal plumose setae, ventral margin with four plumose setae, and one short, simple seta; carpus 1.7 times as long as merus, with six plumose setae and one spine on dorsal margin, and with four plumose setae and four spines on ventral margin; propodus 3.3 times as long as wide, similar in length to carpus, dorsally with one simple and one penicillate seta, and with row of numerous simple setae along ventral and distal margin; dactylus together with unguis 0.7 times as long as propodus, with one dorsal setae.

Pleopods (Fig. 3D) in four pairs, basis with two inner marginal plumose setae; exopod with 13 marginal plumose setae; endopod with 15 marginal plumose setae and proximal acute denticles on inner margin.

Uropod (Fig. 3E) basis 6.5 times as long as wide, with six distal and one marginal setae; exopod of four-segments; segment 2 with two distal setae; last segment with four distal setae; endopod of 13 segments, most with simple setae, last segment with four distal setae.

##### Remarks.

The new species differs from the two previously described species by the length/width ratio of the body, which is 6.7 in *Numbakulla pii* sp. n., 8.0 in *Numbakulla srilankensis* and 4.5 in *Numbakulla pygmaeus*. *Numbakulla pii* also differs in lacking “glandular formations” on the pereopods, present in the other species, having ventral spines on pereopod 6 carpus and acute proximal denticles on endopod of pleopod. *Numbakulla pii* can be distinguished by the appearance of the mandible with a narrow, one-denticled lacina mobilis and palp article 2 with denticles.

The new species is similar to *Numbakulla srilankensis* in having a flat rostrum, well-developed eyes and pereonite 4 clearly longer than the rest, while in *Numbakulla pygmaeus* rostrum is rounded, eyes are absent and all pereonites are similar in length. *Numbakulla pii* resembles *Numbakulla srilankensis* also in bearing row of inner setae on cheliped carpus, but in first species the row contain seven setae and in the second species 24. Both species have also a row of setae on basis of pereopod 1 (absent in Australian species)

*Numbakulla pii* shares with *Numbakulla pygameus* an elongated peduncle article 2 of the antenna, which is short in *Numbakulla srilankensis*.

##### Distribution.

The species is known from Lamont Reef and Sykes Reef, the Capricorn Group, southern Great Barrier Reef, eastern Australia and was recorded from a depth range of 12–27m in coral rubble.

### Key to the species of genus *Numbakulla*

**Table:** 

1	Eyes present, pereonite 4 longer than the rest pereonites, cheliped carpus with inner row of setae	2
–	Eyes absent, all pereonites similar in length, cheliped carpus without inner row of setae	*Numbakulla pygmaeus*
2	Antennule outer flagellum with six segments, antenna article 2 elongated, pereopod 6 carpus with spine	*Numbakulla pii* sp. n.
–	Antennule outer flagellum with five segments, antenna article 2 short, pereopod 6 carpus without spines	*Numbakulla srilankensis*

## Supplementary Material

XML Treatment for Numbakullidae

XML Treatment for Numbakulla

XML Treatment for Numbakulla pii
